# Interword and intraword pause threshold in writing

**DOI:** 10.3389/fpsyg.2014.00182

**Published:** 2014-03-25

**Authors:** Florence Chenu, François Pellegrino, Harriet Jisa, Michel Fayol

**Affiliations:** ^1^Laboratoire Dynamique Du Langage (CNRS & Université Lyon 2), Institut des Sciences de l’HommeLyon, France; ^2^Laboratoire de Psychologie Sociale et Cognitive, Centre National de la Recherche Scientifique – Université Blaise PascalClermont-Ferrand, France

**Keywords:** handwriting, development of writing, dynamics of writing, pause threshold, temporally driven approach

## Abstract

Writing words in real life involves setting objectives, imagining a recipient, translating ideas into linguistic forms, managing grapho-motor gestures, etc. Understanding writing requires observation of the processes as they occur in real time. Analysis of pauses is one of the preferred methods for accessing the dynamics of writing and is based on the idea that pauses are behavioral correlates of cognitive processes. However, there is a need to clarify what we are observing when studying pause phenomena, as we will argue in the first section. This taken into account, the study of pause phenomena can be considered following two approaches. A first approach, driven by temporality, would define a threshold and observe where pauses, e.g., scriptural inactivity occurs. A second approach, linguistically driven, would define structural units and look for scriptural inactivity at the boundaries of these units or within these units. Taking a temporally driven approach, we present two methods which aim at the automatic identification of scriptural inactivity which is most likely not attributable to grapho-motor management in texts written by children and adolescents using digitizing tablets in association with *Eye and Pen*^©^ (Chesnet and Alamargot, 2005). The first method is purely statistical and is based on the idea that the distribution of pauses exhibits different Gaussian components each of them corresponding to a different type of pause. After having reviewed the limits of this statistical method, we present a second method based on writing dynamics which attempts to identify breaking points in the writing dynamics rather than relying only on pause duration. This second method needs to be refined to overcome the fact that calculation is impossible when there is insufficient data which is often the case when working with young scriptors.

## INTRODUCTION

In this paper we are interested in the dynamics of writing. Part of the originality of our approach resides in the fact that we consider the writing of words in the framework of text production. Writing words in real life involves setting objectives, imagining a recipient, translating ideas into linguistic forms, managing grapho-motor gestures, etc. Understanding writing requires observation of how it occurs in real time. Analysis of pauses is one of the preferred methods for accessing the dynamics of writing and is based on the idea that pauses are behavioral correlates of cognitive processes (Schilperoord, 1996). Behavioral manifestations of writing are scriptural activities (movements and pressure of the pen, writing rates) and scriptural inactivity (pauses). The variables that one can examine are diverse and include what was written, what is being or will be written, as well as pauses, writing rates, pressure of the pen and location of these phenomena (e.g., in relation to linguistic units). The first difficulty arises with the fact that researchers tend to use the term *pause *with the assumption that it is always a behavioral correlate of cognitive processes. One other major difficulty resides in the fact that the same cognitive processes may have different behavioral correlates (the writer could be planning during scriptural inactivity as well as during scriptural activity) and the same behavior might reflect different cognitive processes (pausing could be related to planning as well as to revising) at different times, as well as simultaneously (different cognitive processes could take place in parallel). In addition, a pause that occurs during scriptural activity is not necessarily related to this activity. Thus, there is a need to clarify what we are observing when studying pause phenomena. Furthermore, two approaches can be considered for the study of pauses in written text production. A first approach, driven by temporality, would define a threshold and observe where scriptural inactivity occurs. A second approach, linguistically driven, would define structural units and look for scriptural inactivity at the boundaries of these units or within these units. In the temporally driven approach the units are not defined *a priori* but are expected to emerge, while the linguistically driven approach, which avoids the thorny definition of a threshold, is confronted with the problem of the definition of a unit and cannot investigate whether or not a processing unit emerges from the behavioral traces of writing activity.

In this work we adopt a temporally driven approach and will first address the question of the definition of pauses. We then present two methods which aim at automatic identification of scriptural inactivity which most likely can not be attributable to grapho-motor management in texts collected from children and adolescents using digitizing tablets in association with *Eye and Pen^©^* (Chesnet and Alamargot, 2005).

## PAUSES AS MANIFESTATIONS OF COGNITIVE PROCESSES DURING WRITTEN LANGUAGE PRODUCTION

Observables which are associated with written production are diverse. To what has been written and what is being written, one can add on the one hand, writing activity (movements and pressure of the pen) and on the other, scriptural inactivity (pauses). Because we have access to the finished text, as well as to the on-line data during the production of the text, our analysis must consider what has been written, what is in the course of being written, what is going to be written, as well as pauses, writing rates, pen pressure, and the localization of all these phenomena.

Pauses, as well as pen movements, are considered as behavioral correlates of cognitive processes. However, an obstacle resides in the fact that a particular cognitive process may reveal different behavioral correlates, and a particular behavior might display different cognitive processes, at different moments as well as at the same moment (Schilperoord, 2002). In addition, a pause that occurs during a certain type of activity is not necessarily related to this activity but could, for example, be associated to an anterior activity (delay, e.g., fatigue, revision), or a posterior activity (anticipation).

Cognitive processes, whose behavioral correlates we are observing, are not necessarily predicted by theories of writing. The main types of cognitive processes identified as correlates for pauses are *retrieving*, *planning*, *formulating*, *monitoring*, *repairing* (Flower and Hayes, 1981; Levelt, 1989). Apart from the fact that these cognitive processes are very general and are composed of a number of subprocesses, other determinants of pauses have been identified: writers may be thinking of something else (what they are going to do after the task, a shopping list, their next holiday, etc.) and they may even be doing something quite unrelated to the task (scratching their head, adjusting their position on the chair, looking out the window, pushing their glasses up, etc.). Some of these behaviors could be identified through video but you still could be devoted to the task while, for example, scratching your head. Behaviors related to the task are complex and can include, for example, rereading the text produced so far (Alamargot, 2005; Chesnet and Alamargot, 2005; Alamargot et al., 2006, 2007, 2010). One might also imagine that factors in the experimental situation might interfere with the observation of pausing behavior. Finally, behaviors related to the task, but not necessarily underlying cognitive processes, might appear in the data as pauses (for instance, when the scriptor moves the pen for a line return, or when she/he experiences muscle fatigue, cramps, etc.). Thus, pause behaviors can reflect cognitive processes related to the task or not, and can reflect cognitive, physical or socio-psychological causes (Schilperoord, 2002, following De Beaugrande, 1984).

## HOW TO DEFINE A PAUSE?

The term itself is deluding. Ordinarily, a pause refers to a temporary halt, a break in activity or a moment of rest. In what follows, we continue to use the term *pause* even if it is not completely satisfactory simply to refer to what can be observed, making no claim about associated cognitive processes. We determine a pause as a time of scriptural inactivity. In other words, the pen is up, or it is down but not moving (e.g., everything other than down and moving). Even this simple definition poses a problem: the pen may be up, mobile or stationary and correspond to a pause, or it may be down and stationary or slightly moving and correspond to a pause.

A particularly difficult problem is the determination of the time of inactivity, or a threshold. For spoken language, the definition of this threshold is made by reference to perception. The standard threshold of 200–250 ms (Goldman-Eisler, 1968; Grosjean and Deschamps, 1975) is motivated by the fact that pauses that are shorter are not easily perceived (Zellner, 1994). For written language, the most used criterion is a threshold of 1 s (Schilperoord, 2002) or more (Alves et al., 2008, 2012; Olive et al., 2009) for adults. However, some researchers, such as Olive and Kellogg (2002) adopt a threshold of 250 ms in a text composition task. While there is no precise reason advanced for the thresholds of 1 or 2 s, Olive and Kellogg (2002) defend the threshold of 250 ms because this duration corresponds to the time necessary for putting a dot on an *i*, an accent on a letter or a bar across a *t*. One can ask to what extent these thresholds are relevant. For spoken language, the fact that pauses under a certain threshold are difficult to perceive is only relevant for reception and less for production. If pauses are considered as traces of message elaboration, one can not ignore them on the basis that they are not perceptible. For written language, using as a threshold the time necessary to draw a bar across a *t* or for performing a line return has a certain relevance as these behaviors reflect grapho-motor operations which require a certain time but do not necessarily interest researchers examining text production. However, nothing *a priori* allows us to affirm that a pause of this approximate size is not a trace of message elaboration when it does not correspond to a line return or a letter completion (*t *bar, accent, etc.). One can also object that even when a pause corresponds to a grapho-motor operation, it could be the occasion for conducting another activity in parallel, a cognitive one, which would not necessarily slow-down the execution of the grapho-motor operation (in compliance to what is implied by the capacity theory; Just and Carpenter, 1992; McCutchen, 1996).

Consequently due to these objections, a number of questions arise. Is it possible to assign an objective value to a threshold? Should the threshold be the same for all writers? This question is particularly important when studying young writers who may not completely master grapho-motor activity (GMA). Retaining a threshold corresponding to a line return is not as simple as it seems as one can show that the mean time for this type of operation varies across ages. In our data, for example, the shortest time for a line return is 399 ms for the 14–15-year-olds (3^ème^, i.e., 9th grade).; 459 ms for the 12–13-year-olds (5^ème^, i.e., 7th grade); and 615 ms for the 10–11-year-olds (CM2, i.e., 5th grade). This threshold might also depend on the type of task (writing of isolated words, sentence completion, text composing; guided/elicited/spontaneous production, etc.).

Pause analysis raises the crucial question of the definition of a threshold. Two different approaches can be considered: a temporally driven approach and a linguistically driven approach. The temporally driven approach consists of defining a threshold and observing pause locations. The linguistically driven approach consists in postulating the existence of linguistic units and examining how pause duration varies at the boundaries or within these units (Schilperoord, 1996). We consider that both approaches could contribute to the study of writing dynamics. In the temporally driven approach, units are not defined *a priori* but are supposed to emerge from the results of the analyses. The linguistically driven approach permits avoiding the thorny question of a threshold but postulates that structural units as established by linguistic theory constrain the on-line functioning of production.

Determining a threshold leads to excluding from the analysis part of the pauses. Most often excluded pauses are those associated with what is considered motor activity which are not relevant to cognitive processes. Pauses that have physical causes are reputed to be brief (Schilperoord, 2002:75) and are generally considered irrelevant for the analysis of cognitive processes, hence the thresholds of 1 or 2 s most commonly used with adults.

For beginning writers, GMA is not yet or not yet completely automatized. It has been argued that the cognitive load related to GMA in these writers does not allow them to engage in different types of activities in parallel while writing (Chanquoy et al., 1990; Bourdin and Fayol, 1994, 2002; Lambert and Espéret, 1997; Olive and Kellogg, 2002; Peverly, 2006; Alves et al., 2012). Analysis of pauses produced during the course of writing by this type of population requires a methodology that takes into account the specificities of young writers.

In this paper we present, within the framework of a temporally driven approach, two methods for identifying a threshold for cognitive pauses (CP). The reader should bear in mind that as we don’t get to know for our corpus which pauses are cognitive and which are not, the evaluation of each method and their comparison is not straightforward. The first method seeks to establish statistically adaptive thresholds. The results of this first method led us to the second method which attempts to take into account the fact that our writers are of different levels. The second method attempts to detect breaking points in scriptural dynamics. The main objective of both methods is to automatically determine a threshold, to exclude pauses due to GMA and to retain CP only, taking into account the fact that our writers are young scriptors.

## DATA

**FIGURE 1:**
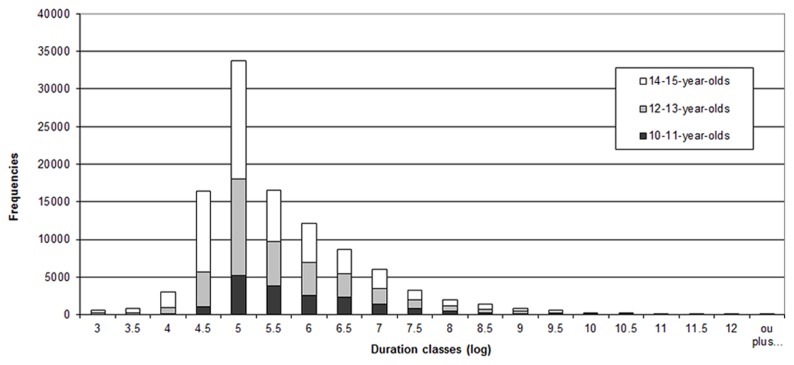
**Pause distribution (log transformed) for all draft texts**.

We dispose of data from three school level gender-balanced groups, each composed of about 40 French-speaking children and adolescents (10–11-year-olds; 12–13-year-olds; 14–15-year-olds). Data collection was conducted using digitizing tablets and the *Eye and Pen* software^1^ (Chesnet and Alamargot, 2005). We did not use video. Each subject produced a narrative text and an expository text in their native language, with a 1 week time interval between the two written productions. For the narrative text, subjects were asked to write a personal implying problems between people. For the expository text, they were asked to write what they know about problems between people (For more details, see Jisa and Viguié, 2005; Maggio et al., 2012). In this study the narrative and expository texts have been combined, our objective being to examine how to tear appart pauses that reflect cognitive processes as opposed to pauses that do not. For each text, a draft and a final version were asked from the participants in compliance with teachers’ usual requirements. Only the draft versions have been used for the current study. A rapid exploration of the final versions reveals that they bear very peculiar patterns of pause distribution, probably corresponding to the copying of information from the draft. The corpus for this study is composed of 278 drafts (90 texts from 10- to 11-year-olds; 94 texts from 12- to 13-year-olds and 94 texts from 14- to 15-year-olds). A minimal threshold of 15 ms has been used for the extraction of pauses in *Eye and Pen*. This very low threshold is the minimum possible in the software and guarantees that relevant pauses are not eliminated *a priori*. Our corpus of draft texts comprises 107 319 pauses (15 ms threshold). **Figure 1** presents the distribution of pauses in all the draft texts. A log transformation has been applied to allow graph representation on the page and to normalize the data (the log transformation of 1 s gives 6.9; 500 ms, 6.2; 200 ms, 5.3). This distribution shows important variations, with a mean pause duration of 760 ms (Median = 147; SD = 5,004).

We observe an important number of short pauses (inferior to 200 ms) and a very asymmetrical distribution which leads to the hypothesis that this is the result of a mixture of different underlying components, potentially corresponding to different types of pauses. This allows us to consider a statistical treatment for the identification of thresholds, as we will present in the next section.

## GAUSSIAN MIXTURE MODEL

The hypothesis under which pause duration reveals their nature leads to considering that the pause distribution in our corpus includes different components (corresponding to different types of pauses). To identify these components, after a log-transformation of the duration for normalization, we used a Gaussian mixture model (GMM), estimated by the Expectation–Maximization algorithm (on Matlab by Igor Cadez 01/23/99) on the set of pauses in each text. The main assumption is that, for a given type of pause, durations are normally distributed (Gaussian), and the goal is thus to identify the statistical parameters (mean and standard deviation) corresponding to each component in the mixture. GMM provides both a statistical description of each component and a classification rule for each pause sample, based on a maximum likelihood decision that can also be seen as a set of duration thresholds based on the statistical distribution.

The main challenge is to set *a priori* the number of Gaussian components underlying the distribution. Moreover, since GMM is a statistical model, the estimation of each Gaussian component heavily depends on (1) the corresponding amount of data and (2) the extent to which the underlying phenomena match Gaussian distributions.

**FIGURE 2:**
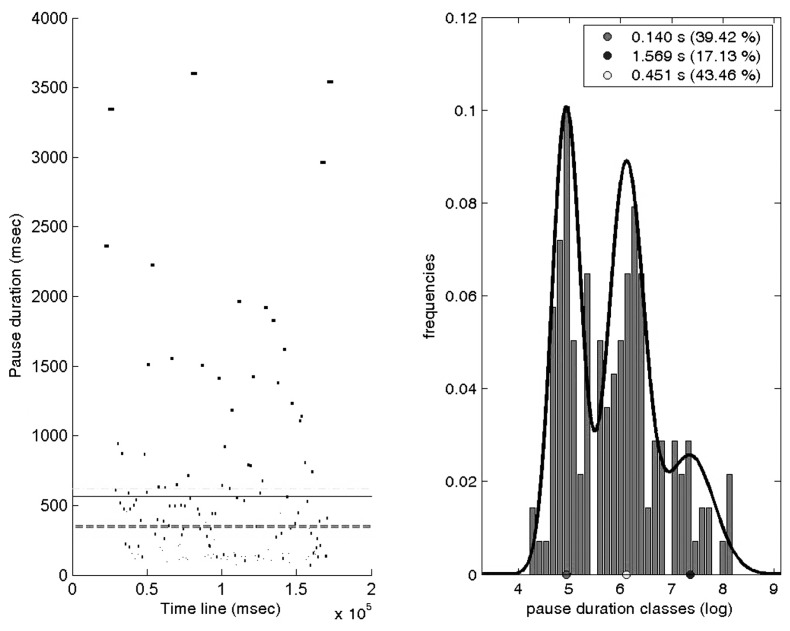
**Pause distribution and Gaussian mixture estimated for the pauses of an expository draft text of a 10–11-year-old subject**.

Even if the theoretical mixture gathers two types of pauses (grapho-motor and cognitive), a careful examination of the data shows that the resulting distribution is not always bimodal, as illustrated on **Figure 2**.

**Figure 2** shows the distribution of pauses and the Gaussian mixture estimated for the pauses observed in an expository draft text of a 10–11-year-old scriptor. On the left side, the horizontal lines represent the overall pause duration mean (gray plain line), median (dark gray dotted line), and 75th percentile (light gray dotted line). The right side shows the duration histogram (which is hardly bimodal) and the corresponding estimation of a Gaussian mixture of three components (black line). The mean value identified for each component appears in the boxed text in the superior right corner with the percentage of pauses that the component integrates. In this example, the algorithm yielded a first component with a mean of 140 ms and including 39.42% of the pauses of this text, a second one with a mean of 451 ms and including 43.46% of the pauses of this text, and a third one with a mean of 1,569 ms and including the remaining 17.13% of the pauses. A graphical comparison between the Gaussian components and the underlying data shown by the histogram suggests that, while the two first components may be normally distributed, the third one seems to be more irregular.

After having computed for each text a set of GMMs with 2, 3, and 4 components, a manual inspection reveals that the optimal number of components depends on the writer and text. The conclusion is that, even if the GMM components may be automatically estimated for each text/writer, the number of components has to be set manually, failing to provide a fully unsupervised algorithm.

It is likely that this method is not well suited for our data, in particular insofar as the texts have been produced by subjects with very different levels of expertise (5th, 7th, and 9th grades). Indeed one can imagine that the types of pauses for a writer who has not fully automatized GMA are different from those of a writer fluent in graphic gestures. The fact that GMA is not yet completely automatised in 10–11-year olds (5th grade) might partially explain the diversity of the results obtained with GMM method. This led us to question how to take into account the level of automatisation of GMA in our subjects.

## ANALYSIS OF MEAN WRITING SPEED

**FIGURE 3:**
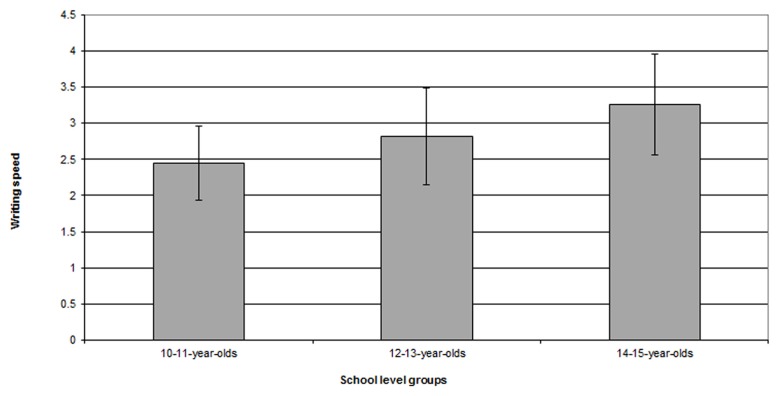
**Mean writing speed in draft texts as a function of school level groups**.

Presumably, differences in the level of automation of GMA will show in the execution velocity of the pen movements. The analysis of mean writing speed should allow for detecting differences between our school level groups (10–11-year-olds, 12–13-year-olds, 14–15-year-olds). To proceed with this analysis, we worked with the pen-down event speeds (excluding down pauses) given by *Eye and Pen*. This measure is displayed in centimeters per second. **Figure 3** presents mean writing speeds (in centimeter per second in moving events, i.e., excluding pauses) as a function of school level groups.

A one factor ANOVA confirms that the mean writing speed increases with school level [*F*_(2,275)_ = 38.38; *p* < 0.0001]. The 10–11-year-olds are slower than the 12–13- and the 14–15-year-olds (*p* < 0.0001), and the 12–13-year-olds are slower than the 14–15-year-olds (*p* < 0.0001). This result strengthens the validity of defining a threshold for each age group, and perhaps even for each subject, given that important variations appear within the groups. In addition, since the older writers write faster, they need less time to move from one word to the next. The younger writers require more time to cover the same distances. A pause of 200 ms between words, then, would not indicate the same operation for a young and older writer. We can argue that the length of a pause – which is not a behavioral correlate of cognitive processes – might well be a function of mean writing speed. The idea then, rather than analyzing all pauses, is to develop a method that allows for the detection of breaking points in the scriptural dynamics.

## DETECTION OF BREAKING POINTS IN THE SCRIPTURAL DYNAMICS

Rather than considering the value of pause duration to estimate its nature, we will attempt to identify real disfluencies, that is to say pauses that create a breaking point in the course of the writing dynamics. In other words, for each event corresponding potentially to a cognitive pause (pen up or not moving), we try to determine if the duration of this event corresponds to the time it takes for that subject to move his pen from the last writing point (end of a word, punctuation, etc.) to the next. In this case, this event is probably not a cognitive pause, even if the duration is quite long. As an example, consider line returns: they need a certain amount of time to be achieved, just by the fact that they require moving the pen from the right of the page to the left. The same is true between words: you need to lift the pen to make a space between words and this takes some time. Hence these events might appear as pauses in the data whereas they are not pauses reflecting cognitive activity.

**FIGURE 4:**
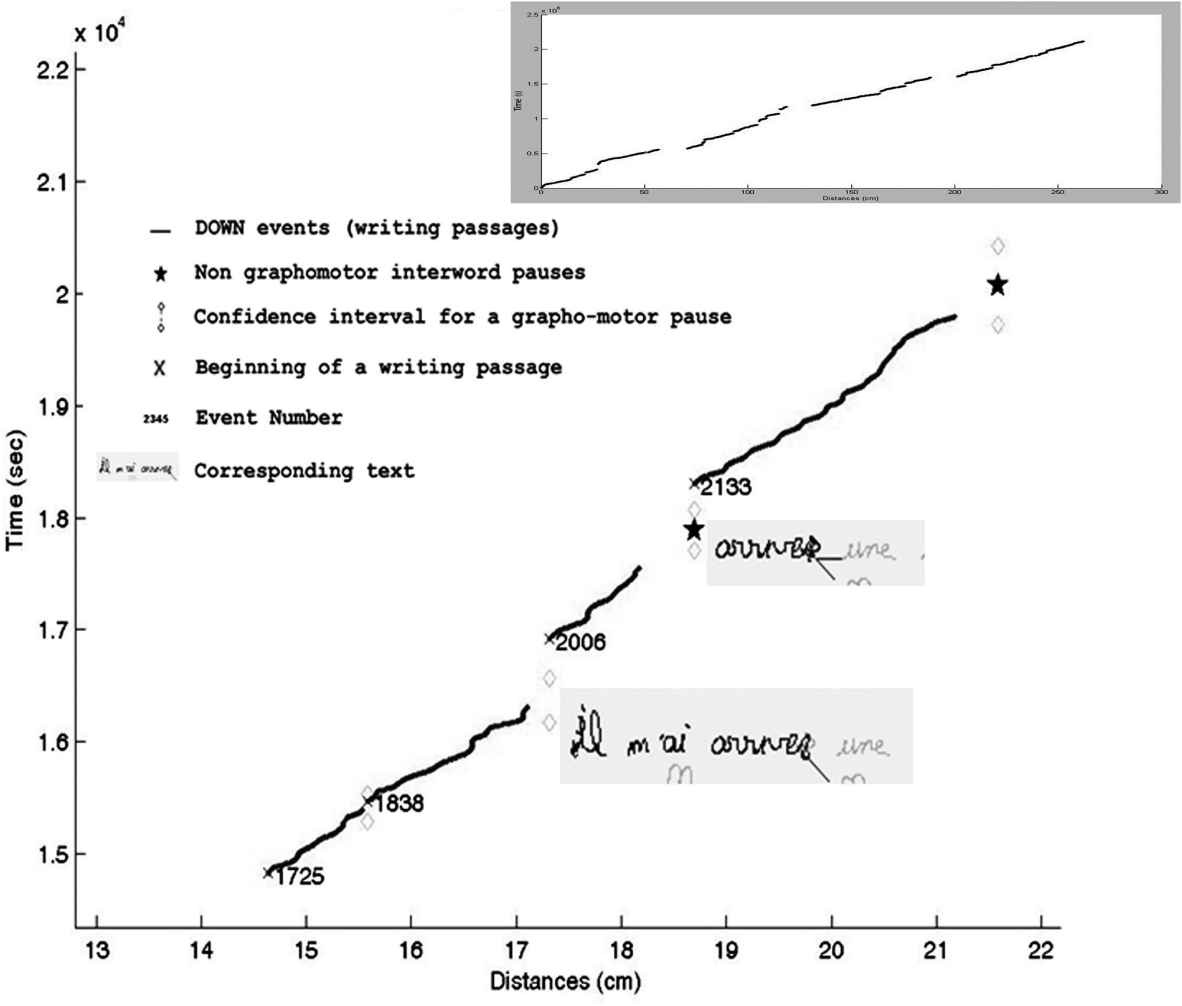
**Detail of the representation of the dynamics of a text**.

The method involves several steps. We first calculate the writing speed of each event for each text. This calculation is undertaken using the temporal and spatial information obtained with the *Eye and Pen* extraction of all events. Events here should be understood as defined by the software, i.e., an event is a sample non-redundant with the preceding (e.g., *Eye and Pen* considers that the data arriving from the digital tablet is of the same event if it has the same pen pressure but if pen pressure changes data is considered as a new event. Each event is identified by a number, a beginning time and location and an ending time and location, a pressure, the distance between beginning and ending locations, and the duration of the event). The second step consists in identifying writing passages (series of events where the pen is down and moving: DOWN) as opposed to pauses (events where the pen is up or not moving: UP). A writing passage (DOWN) is hence a collection of successive events where the pen is touching the tablet. A pause (UP) is constituted of only one event, in which the pen is not touching the tablet. The third step involves using the *Matlab linear prediction* tool to calculate, at each pause event (UP), the expected spatio-temporal position (and a confidence interval^2^) for the beginning of the next writing passage, given the rhythm of the preceding writing passage, under the hypothesis that only GMA is involved. If the event beginning the next writing passage is located outside the confidence interval, the pause is considered not to be caused only by GMA. **Figure 4** represents a detail of the dynamics of a narrative text of a 10–11-year-old pupil the duration of which is 3 min and 30 s with a mean writing speed of 2 cm per second. The dynamics of the whole text is displayed in the right upper box. In this box, each DOWN event is displayed on the graphic by a point. Discontinuities represent pauses. The *y* axis indicates temporality, while the *x* axis represents distance. Lines that tend to be horizontal illustrate accelerations while those with large slopes represent reductions in speed.

Main part of **Figure 4** presents a detail of the representation of the dynamics of a text. Black lines represent writing passages. Note that a writing passage does not necessarily correspond to a word: the pen might be lifted during the writing of a word for more than 15 ms (e.g., to put a bar on a t or because of spelling hesitation) resulting in several writing passages for a word. Events in-between these writing passages are intra-word pauses. A small cross is positioned at the beginning of each writing passage. Diamond shapes indicate the limits of the confidence interval for a motor pause. A non-motor pause can be recognized by the fact that the cross at the beginning of the writing passage is situated outside the confidence interval delimited by the diamond shapes. A black star is plotted when the pause is located between two words and is most probably not a grapho-motor pause. The text corresponding to writing passages is presented in light green boxes. Numbers correspond to the event number of the pauses preceding writing passages. Thus, at event number 2006, the writer has already written *arriver *(“to come”). During the next writing passage, he crosses out the *r*. The pause that takes place at event number 2006, according to our method is identified as a pause which is most probably not caused by motor activity only. At event number 2133, the writer has already written *arriver* and crossed out the *r*, and during the next writing passage he writes *une *(“a/one”). According to our method, the pause that occurs in 2133 is an interword pause and is most probably not caused by GMA only. Note that, with this method, a line return, which always takes time, would be considered as caused by GMA if it stands in the same dynamics as the preceding writing passage.

**FIGURE 5:**
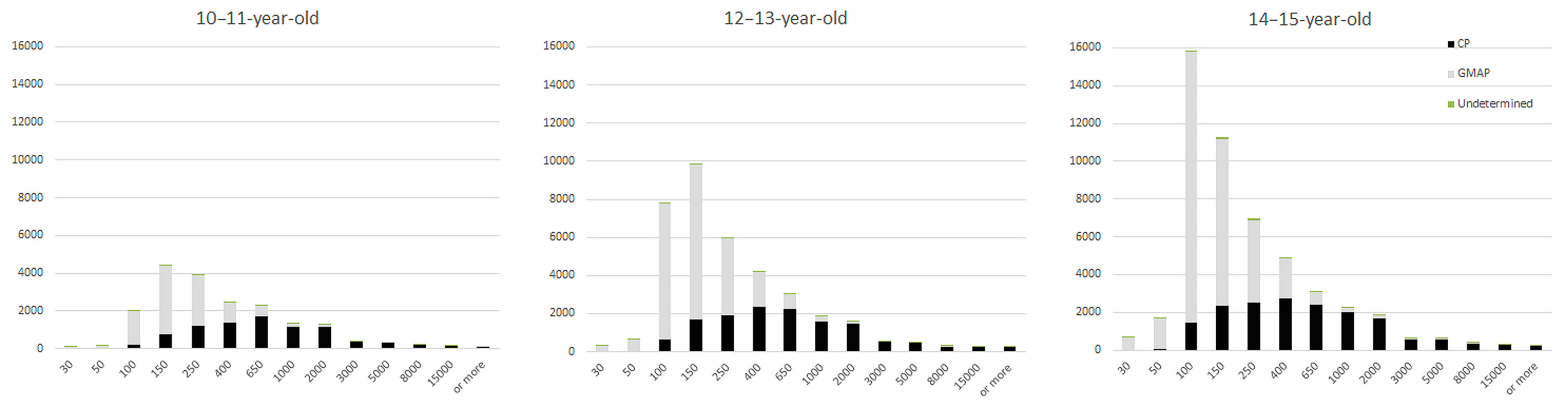
**Pause types identified in 10–11-year-old, 12–13-year-old, and 14–15-year-old texts**.

**Figure 5** presents the distribution of types of pauses for the three age groups. Pauses identified as falling under GMA appear in gray on the graphs, whereas CP are displayed in black. Pauses to which none of these two categories could be assigned (undetermined) are so rare that they are hardly visible on the graphs.

By taking into account the dynamics of scriptural activity, our method excludes a number of short pauses (inferior to 200 ms), in accordance to the commonly admitted idea that short pauses have physical, and not cognitive, causes.

**FIGURE 6:**
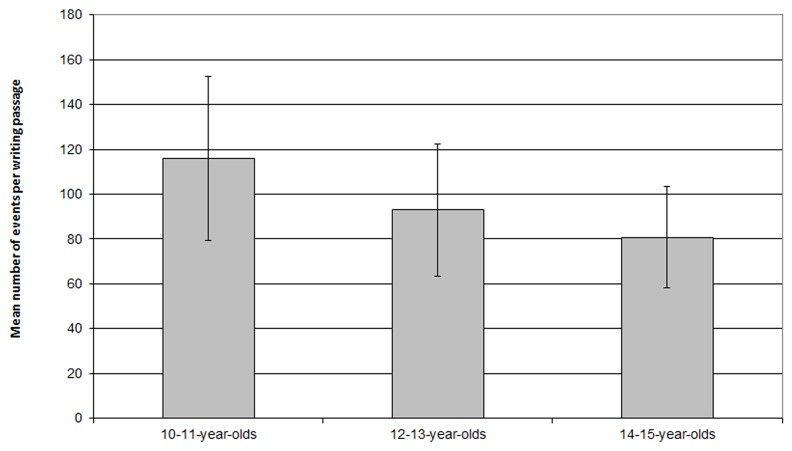
**Mean length of writing passages as a function of school level in terms of number of events**.

Our original goal was to define a threshold for each subject given the different levels of expertise within our groups. This second method involves defining a different threshold for each pause and is strongly motivated, which is not the case with the GMM method. However, this method presents a number of limitations. First, at the level of the calculations, we encounter two main difficulties. The first is an impossibility of calculating when the writing passage before the pause is too short. This occurs when the writer simply puts his/her pen down for a very short time, resulting in very few number of events. This concerns only a few observations, representing up to 5% of the pauses in a few texts. The second is that the calculations do not all have the same level of precision because writing passages are of variable length (in terms of the number of events they include). Each subject presents variation in the length of their writing passages: length of words as well as letters in the words depending on the way these letters are written, and cognitive processes can lead to lifting the pen resulting in different lengths of writing passages. The longer the writing passage is, the better the precision. **Figure 6** presents mean length of writing passages as a function of school level group and shows that the mean length of writing passages is shorter for higher school level groups. This means that with this method, precision is better for lower school level groups than for higher school level groups.

Short writing passages (short series of events where the pen is down and moving) may lead to reject pauses as being grapho-motor despite the fact that they might not be. This limit might be overcome by taking into account the pressure of the pen and down pauses and by weighting the calculation by integrating all writing passages. From a more general point of view, our method does not allow us to exclude extremely long pauses. Regrettably extremely long pauses are too long to be accounted for: they can be due to so many different cognitive processes that other methods should be used to explore them. Furthermore, our method does not identify potential slow-down strategies. Thus, if the writer slows his/her writing rate to free cognitive resources, our method might not detect a breaking point. A writing rate analysis should then be integrated (this would amount to analyzing the derivative of the spatio-temporal curve used here).

The two methods presented here give different results which are not directly comparable. The first method establishes thresholds for each text of each writer whereas the second method established thresholds for each pause of each text of each writer. Furthermore, the number of types of pauses is variable with the first method (it depends on the number of gaussian components identified in the distribution of pauses of each text for each writer) whereas it is fixed with the second method (two types of pauses are considered: pauses most probably explained by GMA opposed to pauses explained by cognitive activity).

## CONCLUSION

Both methods presented here aimed at overcoming the problems inherent in defining a threshold for the analysis of pauses. They are an attempt to take into account the fact that pauses might be other than behavioral correlates of cognitive processes (in particular, they might be caused primarily by GMA) and that a particular pause length may be analyzed differently according to the writer who produces it (beginning versus more experienced writers). If the first method presents important limitations, the method detecting breaking points in the scriptural dynamics has a strong advantage: the categorization of pauses is no longer based on pause length but is motivated by the dynamics of writing. Our results lead us to consider CP as being of shorter duration than frequently used thresholds (in this study the mean length of a pause is 759.81 ms with a median of 147 and standard deviation of 5,004.011). The one second threshold might well be overestimated, even for adult scriptors.

## AUTHOR CONTRIBUTIONS

Expertise in writing dynamics: Michel Fayol, Harriet Jisa; building, testing, and evaluation of the methods: François Pellegrino; Florence Chenu; writing: Florence Chenu; Harriet Jisa; François Pellegrino.

## Conflict of Interest Statement

The authors declare that the research was conducted in the absence of any commercial or financial relationships that could be construed as a potential conflict of interest.

## Footnotes

1 In this data collection the eye tracker device was not used. Only data from the “pen” was recorded.

2 This statistically established interval sets the probability of containing the value to 95%.
